# Adhesive protein-mediated cross-talk between *Candida albicans* and *Porphyromonas gingivalis* in dual species biofilm protects the anaerobic bacterium in unfavorable oxic environment

**DOI:** 10.1038/s41598-019-40771-8

**Published:** 2019-03-13

**Authors:** Dominika Bartnicka, Justyna Karkowska-Kuleta, Marcin Zawrotniak, Dorota Satała, Kinga Michalik, Gabriela Zielinska, Oliwia Bochenska, Andrzej Kozik, Izabela Ciaston, Joanna Koziel, Lindsay C. Dutton, Angela H. Nobbs, Barbara Potempa, Zbigniew Baster, Zenon Rajfur, Jan Potempa, Maria Rapala-Kozik

**Affiliations:** 1 Department of Comparative Biochemistry and Bioanalytics, Faculty of Biochemistry, Biophysics and Biotechnology, Jagiellonian University in Krakow, Krakow, Poland; 2 Department of Analytical Biochemistry, Faculty of Biochemistry, Biophysics and Biotechnology, Jagiellonian University in Krakow, Krakow, Poland; 3 Department of Microbiology, Faculty of Biochemistry, Biophysics and Biotechnology, Jagiellonian University in Krakow, Krakow, Poland; 4 Bristol Dental School, University of Bristol, Bristol, United Kingdom; 5 Department of Oral Immunology and Infectious Diseases, University of Louisville School of Dentistry, Louisville, KY USA; 6 Institute of Physics; Faculty of Physics, Astronomy and Applied Computer Science, Jagiellonian University, Krakow, Poland

**Keywords:** Microbiology, Diseases

## Abstract

The oral cavity contains different types of microbial species that colonize human host via extensive cell-to-cell interactions and biofilm formation. *Candida albicans***—**a yeast-like fungus that inhabits mucosal surfaces**—**is also a significant colonizer of subgingival sites in patients with chronic periodontitis. It is notable however that one of the main infectious agents that causes periodontal disease is an anaerobic bacterium**—***Porphyromonas gingivalis*. In our study, we evaluated the different strategies of both pathogens in the mutual colonization of an artificial surface and confirmed that a protective environment existed for *P. gingivalis* within developed fungal biofilm formed under oxic conditions where fungal cells grow mainly in their filamentous form i.e. hyphae. A direct physical contact between fungi and *P. gingivalis* was initiated via a modulation of gene expression for the major fungal cell surface adhesin Als3 and the aspartic proteases Sap6 and Sap9. Proteomic identification of the fungal surfaceome suggested also an involvement of the Mp65 adhesin and a “moonlighting” protein, enolase, as partners for the interaction with *P. gingivalis*. Using mutant strains of these bacteria that are defective in the production of the gingipains**—**the proteolytic enzymes that also harbor hemagglutinin domains**—**significant roles of these proteins in the formation of bacteria-protecting biofilm were clearly demonstrated.

## Introduction

*Candida albicans*, a commensal yeast-like fungus that commonly colonizes human mucosal surfaces^1^ often becomes an opportunistic pathogen in immunocompromised patients, thereby causing recurrent mucosal infections or life-threatening disseminated infections with high mortality rates^2^. During colonization or infections, *C. albicans* adheres to host surfaces and exploits an ability of fungal cells to switch their morphology between yeast and hyphal forms. The latter form promotes the formation of a structured microbial community, the biofilm, that increases a resistance of the fungal cells to antimicrobial agents or to environmental changes^3^. Notably also, the microbial biofilm formed by *C. albicans* can consist not only of this single fungal species but also contain numerous different species of bacteria, depending on the affected host niche^4^.

The oral cavity contains the highest diversity of microorganisms among all human microbial habitats^5^ and encompasses two types of functional surfaces, the mucosal surface and the teeth. Moreover, the various sites of the oral cavity can represent different conditions in terms of nutrient and oxygen availability The microorganisms that are early colonizers of the salivary pellicle on the tooth surface are streptococci, with *Streptococcus mutans* as the main bacterial pathogen involved in dental caries^6^ and some bridging microorganisms such as *Fusobacterium nucleatum*^7^. Both can be involved in the interactions with hyphal filaments of *C. albicans*, promoting co-colonization of these surfaces by yeast^8^.

Notably however, biofilm that extends below the gum line becomes subgingival plaque, where more pathogenic, Gram-negative anaerobes such as *Porphyromonas gingivalis* can arise^9^. *P. gingivalis* is the main infectious agent of periodontal disease, which involves an inflammatory process with many states and stages, ranging from easily curable gingivitis to irreversible severe periodontitis^10^. Moreover, it is believed that these bacteria play major roles as etiological factors or synergistic contributors in the multifaceted stimulation of the host immune system that can tip the scales towards development of chronic or autoimmune pathological states such as heart diseases, respiratory diseases, the osteoporosis or arthritis^11^.

Patients with chronic periodontitis show a significantly higher level of colonization with *Candida* spp. at subgingival sites than healthy individuals^12^. Moreover, *C. albicans* has been also frequently isolated from periodontal pockets^13–16^. It has also been reported that some *C. albicans* genotypes are unique to subgingival isolates, an observation that may reflect a selection process for *C. albicans* cells that are better adapted to this more anaerobic environment or to a coexistence with pathogens in plaque biofilm associated with periodontitis^17^.

Despite the extensive exploration of fungal-bacterial coexistence in prior studies, research concerning the interactions of *C. albicans* and *P. gingivalis*, particularly in the context of polymicrobial infections, is still at its infancy and in the context of periodontal disease has been nearly completely overlooked. Nevertheless, recent studies suggest that *C. albicans*, residing in different niches of the host organism, creates biofilms that provide a hypoxic microenvironment inside the biofilm community and support the growth of some anaerobic bacteria, even under apparently aerobic conditions that are normally toxic to them^18^. A gradient of oxygen concentration down to ca. 4% has been observed in *C. albicans*-formed biofilm, confirming the heterogeneous nature of this structure. In turn, *P. gingivalis* in a continuous culture system, depending on hemin availability, can tolerate low levels of oxygen (6–10%), and still reach steady-state growth. However, some of the physiologic processes of *P. gingivalis* have been found to be modulated^19^, thereby documenting a quick adaptation of bacteria to these new conditions. Moreover, *C. albicans* cells can survive and form biofilms under anaerobic and nutrient-limited conditions, and thus may pose a treatment challenge^20^. Another mechanism for protection of anaerobic bacteria by *C. albicans* cells was proposed for *Clostridium difficile* by Leeuwen and coworkers^21^ who suggested a reduction of oxygen level in the microbial environment as the consequence of antioxidant production or metabolic changes of *C. albicans* cells contacting these bacteria. However, in such microbial community the formation of fungal biofilm is suppressed and the bacterial adherence to *C. albicans* hyphae, rarely formed, is limited to their tips.

In the light of these data, we here examined a possible protection of the gingival anaerobe **-** *P. gingivalis* **-** through the formation of mixed biofilm with *C. albicans* inside which the bacteria can find better conditions for survival. We identified also the main bacterial and fungal surface proteins involved in mutual microbial interactions between these two potentially dangerous colonizers of the human oral cavity.

## Results

### Viability and adhesion properties of fungal (*C*. *albicans*) and bacterial (*P*. *gingivalis*) cells during the formation of different types of biofilms

To identify biofilm-favoring conditions, three different models of microbe contact were applied. In first two (sequential) a single-species biofilm (either fungal or bacterial) was developed prior to its colonization by the second microorganism. This model simulated different pathological conditions that occur during periodontal infections, where a dominant biofilm can be colonized by a second invading microbe. The third model (simultaneous), which was probably less likely to arise *in vivo*, involved the common contact of both pathogens that competed or cooperated in the colonization of the new surface. In all models, both anoxic or normoxic conditions were considered. In addition, as gingipains belong to the main virulence factors for *P. gingivalis*, we also compared the response of a wild strain of these bacteria with mutant strains lacking the genes that encode these proteolytic enzymes.

**Figure 1:**
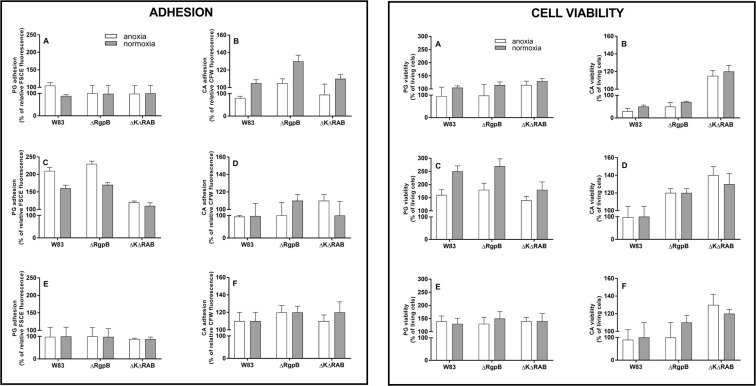
Adhesion (left panel) and viability (right panel) of *P. gingivalis* (PG) and *C. albicans* (CA) cells that form a dual-species biofilm (**A**,**B**) *Sequential model type I*. A biofilm was formed by CFSE-stained bacteria for 24 h at 37 °C in Schaedler medium under anoxic conditions and this mono culture was covered with *C. albicans* cells and further propagated for 3 h at 37 °C in RPMI medium under both anoxic and normoxic conditions. After biofilm washing, the fungal cells were visualized by CFW staining. Adhesion was determined fluorometrically. Cell viability was determined after the biofilm had formed by cell scratching and propagation on the agar plate in media and under conditions appropriate for each species. (**C**,**D**) *Sequential model type II*. A biofilm was formed by *C. albicans* cells for 24 h at 37 °C in RPMI medium under normoxic conditions, and then settled with bacteria for 3 h at 37 °C in RPMI medium under anoxic and normoxic conditions. Further analyses were performed as described in (**A**,**B**). (**E**,**F**) *Simultaneous model*. A dual-species biofilm was formed by bacterial and fungal contact at the cell surface at the same time for 3 h at 37 °C in RPMI medium under anoxic and normoxic conditions. Further analyses were performed as described in A and B. All experiments (A–F) were performed with five repetitions in triplicate. The data shown are relative to a mono-species biofilm propagated under the same conditions. Data were presented as means ± SD (standard deviation) and analyzed using GraphPad Prism software (GraphPad, LaJolla, CA).

We first considered the bacteria as the initial surface colonizer that formed a stabile biofilm for 24 hours under anaerobic conditions and then enabled contact with *C. albicans* cells in the presence or absence of oxygen (Fig. 1, both panels, A, B). The stability of the bacterial biofilms formed by wild or mutant strains was unaffected by *C. albicans* colonization under both anoxic and normoxic conditions (left panel, A). However, bacterial biofilm viability increased by 20% in the presence of *C. albicans* cells in a normoxic environment (right panel, A). The viability of fungal cells was reduced to 35% by contact with the *P. gingivalis* wild type strain biofilm under anoxic conditions that favored bacterial growth and total gingipain activity (right panel, B). This observation suggests a dominant role of gingipains in mixed biofilm creation, where the proteolytic activity as well as agglutinin properties of these proteins could be essential. A reduction of gingipain activity in *P. gingivalis* cells by elimination of RgpB (∆RgpB) resulted in a 35% increase of fungal cell adhesion at normoxia (left panel, B). On the other hand, the elimination of all gingipains (∆K∆RAB) influenced mainly the fungal cell viability at both type of conditions but the adhesion, especially under anoxia was lower compared to the contact with wild type bacteria. It can be the first evidence for the importance of agglutinin domains of RgpA and Kgp in such interactions.

In the second model, where the fungal biofilm was developed under aerobic conditions for 24 h prior to contact with bacterial cells, with a domination of hyphal form of fungal cells, the bacteria exerted a marginal impact on them. However, wild type *P. gingivalis* under anoxic conditions lowered the fungal adhesion to the artificial surface, probably due to a degradation of fungal surface proteins (left panel, D). Nevertheless, bacteria were able to positively modulate the fungal viability, with the most pronounced effect observed during their contact under anoxic conditions with mutant strains defective in active gingipain production (right panel, D). On the other hand, bacteria presented more beneficial effects towards the fungal surfaces in terms of adhesion properties and viability (c.a. 200% increase), which were observed under anoxic and normoxic conditions for the wild type strain and also for the mutant strain partially lacking proteolytic activity (∆RgpB). However, no significant adhesion or viability enhancement was observed for the mutant lacking all gingipain activities and adhesive domains of RgpA and Kgp, again indicating the strong interactions between gingipains and fungal compounds located on the hyphal surface (both panels, C).

The findings for the third model of microbial co-adhesion, where both microorganisms contacted the artificial surface at the same time, corresponded to those obtained for the developed fungal biofilm under normoxia, with a protective effect of fungal biofilm on bacterial cell viability (right panel, E).

**Figure 2:**
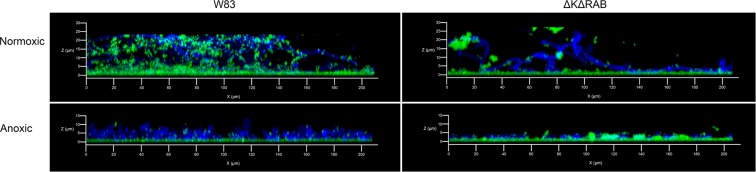
Microscopic evaluation of the *sequential type II* biofilm. The biofilm was prepared on poly-L-lysine coated glass slides as described for Fig. 1. CFSE-stained bacteria (green) and CFW-labeled fungal cells (blue) were incubated for 24 h at room temperature, then fixed with 3.8% paraformaldehyde.

The aforementioned observations concerning microbial adhesion were confirmed by the analysis of the formed biofilms using confocal microscopy (Fig. 2). Under anoxic conditions, the bacterial cells were located mainly at the flat biofilm surfaces. However, during fast hypha development of *C. albicans* at normoxic conditions, the bacterial cells adhered directly to the growing hyphae. This effect was significantly reduced in the case of the mutant ∆K∆RAB.

Taken together, these results indicated that a developed fungal biofilm can serve as a protective environment for *P. gingivalis* and enable these bacteria to survive under unfavorable normoxia conditions. A question from this that remained to be resolved was the identity of the microbial surface components that could be responsible for this effect.

### Expression changes in the genes encoding selected fungal virulence factors in mixed biofilms

The microorganisms that develop into an interspecies biofilm often change the type or levels of the expressed virulence factors employed, due to mutual interactions or regulatory mechanisms. Such changes were observed previously during biofilm formation between *C. albicans* and *Streptococcus gordoni*^8^ or *Streptococcus oralis*^22–24^.

**Figure 3:**
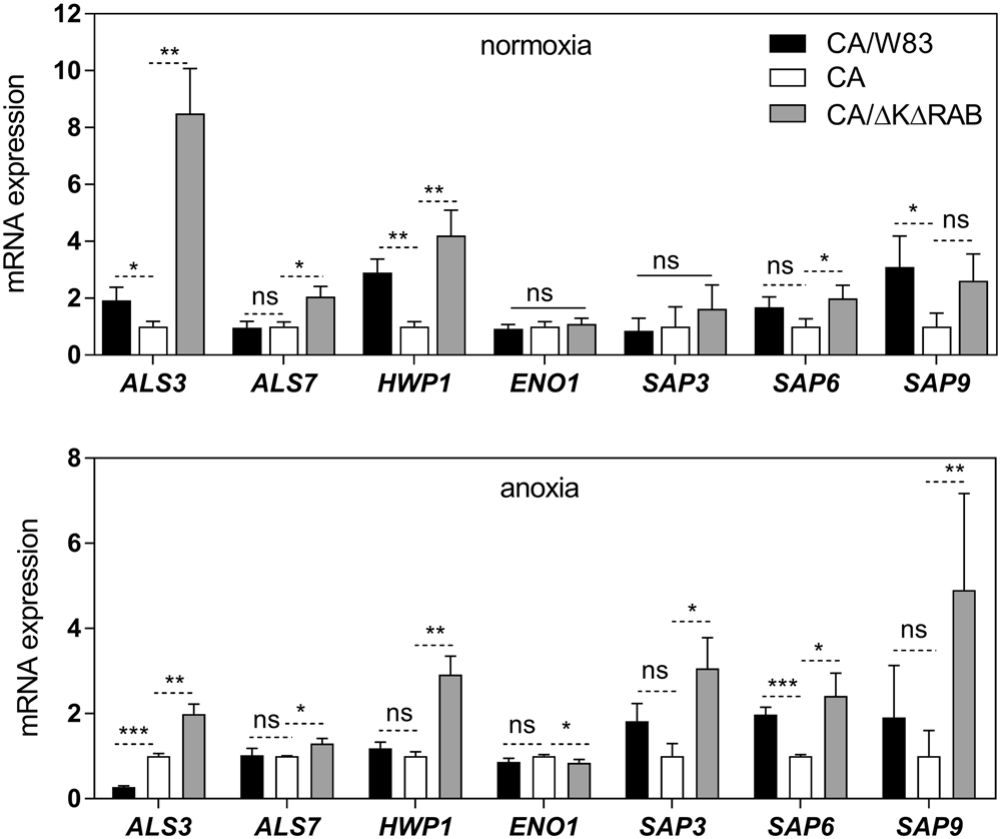
Changes in the expression levels of selected proteinous virulence factors in *C. albicans* during contact with *P. gingivalis*. After a 3 h contact between *C. albicans* and *P. gingivalis* cells in the *simultaneous model* of interaction under anoxic and normoxic conditions, total RNA was extracted from fungal cells using TRI reagent treatment. Real time quantitative PCR was then performed with a SYBR green detection assay. The selected genes encoding fungal surface protein Hwp1, adhesins of the agglutinin-like family, Als3 and Als7, aspartic proteases, Sap3, Sap6 and Sap9, and a cytosolic/‘moonlighting’ protein enolase (Eno1) were analyzed. The C_T_ values (the average threshold cycle) were normalized to that of the *ATC1* gene. To quantify gene expression, comparative C_T_ was used and the relative expression was determined using the 2^−ΔΔCT^ method^71^. Results are representative of three independent experiments and are expressed as means ± SD (standard deviation); n = 3. *P < 0.05; **P < 0.01; ***P < 0.001. The analysis was performed using GraphPad Prism software (GraphPad, LaJolla, CA, USA) with an one-way ANOVA test.

To verify the relevance of these factors in biofilm formation between *P. gingivalis* and *C. albicans*, we analyzed changes in the expression level of the genes encoding representative adhesins belonging to Als family **-** Als3 and Als7, the cell surface adhesin **-** Hwp1, and a “moonlighting” protein ubiquitously expressed on the *C. albicans* cell surface **-** enolase (Eno1)^25^. We considered also the possible involvement of genes encoding proteases known to be preferentially secreted in yeast or hyphal forms of *C. albicans* (Sap3 and Sap6, respectively) and the surface-located and ubiquitously present Sap9^23^. For these analyses, we applied the model of simultaneously formed biofilm after 3 hours of mutual contact under anoxic and normoxic conditions of the wild type *P. gingivalis* strain W83 and its mutant deprived of all gingipains (∆K∆RAB). The data (Fig. 3) showed a significant (10-fold) increase in *ALS3* gene expression, mainly under normoxic conditions, in contact with the ∆K∆RAB mutant of *P. gingivalis*. A similar effect, although with a lower intensity was observed for *HWP1* expression (5-fold increase). In contrast, the genes encoding Sap proteases were more active under anoxic conditions, with 3-fold increase in the expression of *SAP9* encoding gene. Again, this phenomenon seemed to be slightly more intense during the contact of *C. albicans* cells with the mutant strain (∆K∆RAB). The expression of the *ENO1* gene, encoding the essential glycolytic enzyme, enolase, was unchanged during 3 hours of fungal cell contact with any bacterial cells.

### Differences between the fungal surface proteomes in mono- and dual-species (with *P. gingivalis*) biofilms

The significant expression changes identified for genes encoding different virulence factors of *C. albicans* that formed biofilm with *P. gingivalis* led us to investigate the surfaceome of *C. albicans* cells located within a mixed biofilm (Table 1). Under anoxic conditions, where *P. gingivalis* activity dominates, an overproduction of only three fungal proteins was identified compared to the surface proteins exposed in the mono-species biofilm. These were Eno1 (about 11-fold excess), Mp65 (9.4-fold excess) and Eng1 (2.5-fold excess). Although Eno1 is primarily a cytosolic enzyme involved in the glycolytic pathway, it has also been identified among candidal biofilm components^26^. Moreover, it has also been documented^25^ that extracellular enolase is not derived from simple fungal cell lysis but is actively secreted by both the yeast and hyphal forms of *C. albicans* cells, although the mechanism remains unknown. On the other hand, Mp65 is a cell wall mannoprotein of *C. albicans* with putative β-glucanase activity. It harbors multiple glycosylation sites and contains an RGD motif involved in surface adhesion. It is required for hyphal morphogenesis and belongs to the major targets of immune response in humans, similarly to enolase^27^. Eng1 is a cytosolic or periplasm-located protein often secreted into the media but its function outside the cell is unknown^28^.

**Table 1:** Mass spectrometry analysis of *C. albicans* proteins identified at the cell surface after growth in mixed-species biofilm with *P. gingivalis*.

Accession number	Protein name	Normoxia		Anoxia	
		W83	∆K∆RAB	W83	∆K∆RAB
O94049	Acetyl-coenzyme A synthetase 1 (Acs1)	**6.0**	nd	nd	**nc**
P46273	Phosphoglycerate kinase (Pgk1)	**4.6**	nd	nd	nd
P83776	Hexokinase-2 (Hxk2)	**4.4**	nd	nd	nd
P30575	Enolase 1 (Eno1)	**3.0**	**2.5**	**11.2**	**5.4**
P40953	Chitinase 2 (Cht2)	**3.0**	**5.2**	nd	**4.0**
Q5AIR7	Endo-1,3(4)-β-glucanase 1 (Eng1)	**2.6**	1.9	**2.5**	1.4
Q5AD07	Cell surface Cu-only superoxide dismutase 5 (Sod5)	**2.5**	nd	nd	nd
P43076	pH-responsive protein 1 (Phr1)	**2.2**	nd	nd	nd
P0CU38	Agglutinin-like protein 2 (Fragment) (Als2)	2.2	**9.3**	nd	**14.8**
Q59XX2	Cell surface mannoprotein MP65 (Mp65)	1.6	**5.5**	**9.4**	**6.9**
P43067	Alcohol dehydrogenase 1 (Adh1)	1.4	nd	nd	**5.9**
Q5AFA2	Extracellular glycosidase (Crh11)	1.2	1.7	nd	nd
Q59L12	Agglutinin-like protein 3 (Als3)	1.1	**7.0**	nd	nd
Q59TP1	Cell wall protein RTB1 (Rbt1)	1.0	**2.5**	nd	nd
Q5A8T4	Agglutinin-like protein 1(Als1)	0.4	**3.4**	nd	nd
Q92211	Glyceraldehyde-3-phosphate dehydrogenase (Tdh1)	0.2	nd	nd	0.9
Q5AMT2	Glucan 1,3-β-glucosidase (Bgl2)	nd	**3.9**	nd	nd
Q9UWF6	Lysophospholipase 1 (Plb1)	nd	**3.1**	nd	nd
P0CY35	Elongation factor 1-α 1 (Tef1)	nd	nd	nd	**2.7**

5 × 10^8^ *C. albicans* cells were grown with 5 × 10^9^ *P. gingivalis* W83 or ∆K∆RAB cells in RPMI 1640 medium for 24 hours at 37 °C under normoxic or anoxic conditions. Proteins were identified with LC-MS/MS after cell surface shaving with trypsin and additional digestion of released protein fragments for 24 hours. The resulting lists of peaks were used to search against the SwissProt protein database. Protein ranking with indicated fold changes according to the averaged NSAFs was created after comparison of *C. albicans* surface-exposed proteins released from cells grown in mixed-species biofilms to the sample containing fungal surface-exposed proteins of *C. albicans* grown in a single-species biofilm. Markedly enhanced protein presence at the surface of the fungal cells is highlighted in bold (nc, not comparable; nd, not detected).

The fungal surfaceome identified in the biofilm formed by *C. albicans* with a *P. gingivalis* mutant strain bereft of gingipains was also shown to contain a typical agglutinin-like adhesin, Als2 (14.8-fold increase), cytosol-derived alcohol dehydrogenase (Adh1; 5.9-fold increase), chitinase (Cht2; 4-fold increase) and elongation factor Tef1 (2.7-fold increase). All of these proteins were previously identified from candidal biofilm structure^29^.

Under the aerobic conditions preferred by *C. albicans* for growth, the increase in the amounts of proteins listed above was not as significant as at anoxia (2–6-fold), but additional cytosolic proteins were detected (Pgk1, Hxk2), probably resulting from fungal cell degradation by *P. gingivalis* W83 proteases. This possibility was supported by the expression of typical cell wall-damage response proteins, Phr1 and Sod5^30^. Moreover, analysis of the biofilm formed between fungal and ∆K∆RAB cells under normoxic conditions additionally confirmed such a possibility, as in this case we did not observe any changes in the expression of proteins involved in fungal cell wall protection and none of the previously identified proteins typically functioning only in the cytosol were observed. What is also important to note is that a significant increase in the presence of agglutinin-like adhesins Als2, Als3 and Als1 (9.5, 7 and 3.5-fold increase, respectively) was observed compared to mono-species biofilm. These results suggest that these proteins could be involved in the interaction with *P. gingivalis* cells. Notably however, bacterial cells fully equipped with gingipains probably use different or additional fungal proteins during mutual contact within a biofilm. This suggestion comes from the prior observation that Als proteins were diminished in biofilm formed for a prolonged period and seemed to be more potently degraded by bacterial proteolytic enzymes, mainly gingipains (data not presented).

### Confirmation of the interactions between selected candidal cell wall proteins and *P. gingivalis* cells

**Figure 4:**
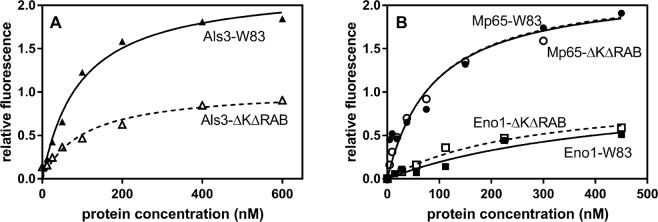
Binding of the fungal surface proteins Als3, Mp65 and Eno1 to *P. gingivalis* cells. Purified and fluorescein-stained fungal proteins were tested for binding to *P. gingivalis* wild type and mutant (∆K∆RAB) strains using FACS analysis. The results are representative of three independent experiments performed in triplicate. The fitting curve was generated using GraphPad software.

On the basis of our results suggesting the importance of the Als protein family, Mp65 cell wall protein and Eno1 in dual-species biofilm formation, we tested for possible interactions of these proteins with the wild type W83 strain of *P. gingivalis* and its mutant strain - ∆K∆RAB, using FACS analysis of bacterial cells after contact with FITC-labeled fungal proteins. The results presented in Fig. 4A showed that Als3 tightly interacts with the cell surface of both *P. gingivalis* strains. The elimination of gingipains however caused a significant reduction in the Als3-binding level. This result clearly indicated an involvement of gingipains in the interaction with fungal adhesin Als3, although other targets for Als3 would necessarily have had to exist.

Two additionally identified fungal proteins, Mp65 and Eno1, also bound strongly to the *P. gingivalis* surface but no difference was observed between the binding properties of the wild type and mutant strains towards both fungal proteins (Fig. 4B). This could indicate that gingipains are not involved in these interactions or that there are other targets for these proteins which compensated for the interactions of the mutant cells.

### Characteristics of RgpA complexes formed with the fungal cell wall proteins Als3, Mp65 and Eno1

**Figure 5:**
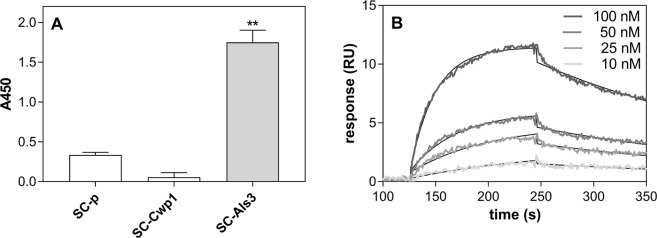
Interaction of RgpA with Als3. (**A**) The interaction of purified RgpA with *S. cerevisiae* containing surface overexpressed Als3 and Cwp1 or an empty pBC542 vector. The results are representative of three independent experiments and are expressed as means ± SD; n = 3. **P < 0.01 (by ANOVA with a Tukey test). (**B**) Interaction of RgpA with Als3 immobilized on the surface of a CM5 chip and analyzed by SPR measurements. The collected SPR data were analyzed using BIA evaluation software version 4.1 (GE Healthcare) using a simultaneous fitting of constants for the association rate (ka) and dissociation rate (kd) to the 1:1 Langmuir binding model with a baseline drift.

To further evaluate the gingipain role in the interaction with fungal biofilm proteins, a purified representative enzyme, RgpA, was used that contains both protease and agglutinin-like domains in its structure. The RgpA interaction with Als3 was first verified by testing its direct binding to *S. cerevisiae* cells with overexpressed Als3 exposed on the cell surface. To eliminate possible nonspecific binding of RgpA to fungal proteins, its binding to *S. cerevisiae* transformed with pBC542 plasmid alone or with cloned yeast *CWP1* was analyzed for comparison^31^. The results of this analysis clearly supported our previous finding that gingipains are the target for Als3 (Fig. 5A). Moreover, the quantitative analysis of the Als3-RgpA interaction was performed, using SPR measurements, where Als3, isolated from the fungal surface was immobilized on the CM5-chip and tested for its interaction with RgpA present in solution poured over the chip surface at an increasing protein concentration range (Fig. 5B). The interaction was characterized by a dissociation constant (Kd) of 9.96 nM, which is strong enough to consider RgpA as an important determinant of candidal interaction with *P. gingivalis* cells within the biofilm structure.

**Figure 6:**
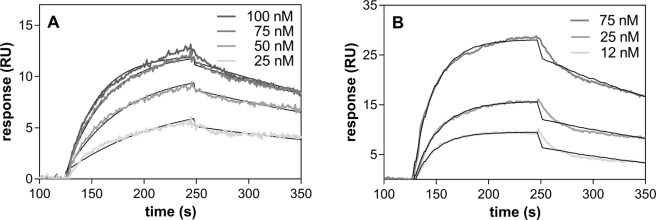
Interaction of the *C. albicans* proteins Mp65 and Eno1 with RgpA. RgpA binding to Mp65 and Eno1 was analyzed by SPR method using *C. albicans* proteins immobilized on a CM5 chip surface (ca. 300 RU) and contacting RgpA at a specified concentration range. Three independent experiments were performed in each case in triplicate. The data were analyzed using BIA evaluation software version 4.1 (GE Healthcare) using the 1:1 Langmuir binding model with a baseline drift.

The role of Mp65 and Eno1, commonly identified as mixed biofilm components, in terms of possible interactions with gingipains was not clear as both wild type and a mutant strain defective in these protease productions interacted to the similar degree with these proteins. Although the targets in both *P. gingivalis* strains could possibly be different, we checked for possible interactions between RgpA and both fungal proteins using the SPR method. Both fungal proteins were bound to the chip surface thereby simulating their natural context within a biofilm. For the classical fungal adhesin, Mp65, the complex formed with RgpA was characterized by a Kd of 9.44 nM, i.e., the binding strength was similar to that for Als3 (Fig. 6A). On the other hand, we surprisingly found that Eno1 bound to RgpA with a 3-fold higher affinity than typical adhesins, with a Kd of 2.97 nM (Fig. 6B). We speculated therefore that a large amount of enolase detected within fungal biofilm could serve as additional support for the interactions between cells in a mixed microbial biofilm. However, the issue of which additional proteins exposed on the surface of the *P. gingivalis* mutant strain could be involved in the interaction with enolase and Mp65, remained to be clarified. The biology underlying this is likely to be quite complex due to the fact that gingipains are also involved in cell surface organization^32^.

## Discussion

The oral cavity is the most diverse niche for microbial species including bacteria and fungi. Moreover, this environment undergoes continuous transformations, depending on the host age (i.e. from first teeth to dentures), diet, variable flow of saliva, pH, oxygen fluctuation, or prolonged use of drugs, especially antibiotics. To survive under such variable conditions, microorganisms develop biofilm communities and use diverse interaction mechanisms to protect or to dominate in these structures^33^.

*C. albicans* belongs to the most common oral fungal commensals. Although detected in oral microflora of healthy hosts in low amounts compared to bacteria, its colonization properties increase significantly in immunocompromised patients, especially if the environment favors the development of a hyphal morphology of the fungus and biofilm formation^34^. At sites of invasive fungal infection, the influx and activity of human host immune cells contribute to the development of hypoxic conditions, to which *C. albicans* cells can be well adapted as has been documented for fungal colonization of the human gastrointestinal tract^35^. Major *C. albicans* hypoxia responses affect the expression of genes producing transcripts, mainly hypha-specific, involved in cell wall and membrane structures, glycolysis and fermentation^36^. The broad adaptation abilities of *C. albicans* enable this fungus to colonize different niches in the oral cavity, creating mixed biofilms with aerobic, intermediate and anaerobic bacteria where mutualistic or commensal relationships are adopted to increase the resistance to the host immune defense or antimicrobial therapy^37^.

Recent evidence has suggested that *C. albicans* cells developing into a biofilm can cooperate with strictly anaerobic bacteria under aerobic conditions, thereby allowing these bacteria to survive and proliferate in an unfavorable environment and possibly induce a disease-associated phenotype^18,38^. Although the mechanisms of such anaerobic micro-niche formation by yeast are still not fully recognized, it was postulated that they could be related to the high level of O_2_ consumption by yeast^39^.

In order to evaluate and verify the different possible modes of microbial coexistence we applied three different experimental models to analyze the relationships between *C. albicans* and *P. gingivalis* cells forming a mixed biofilm. Despite prior evidence for the presence of *Candida* species in subgingival plaques or periodontal pockets^40^, it is difficult to attribute a definitive role for *Candida* spp. in the etiology of periodontitis in which *P. gingivalis* seems to be the keystone pathogen whose adhesion and viability could not be influenced significantly by contacting fungal cells. In contrast*, C. albicans* viability was found to be strongly dependent on the proteolytic potential of *P*. *gingivalis*, regardless of its adhesive properties. Moreover, the joint growth and competition for surface settlement under aerobic conditions favors *C. albicans* cell adhesion and hypha development that supports bacterial cell viability. Under anoxic conditions, preferred by bacteria for growth, fungal cell survival was still found to be dependent on gingipain activity. Contrary to this, when the surface is first settled by *C. albicans* cells that have formed a mature biofilm in which the hyphal form of the fungus is dominant and creates hypoxic conditions within the biofilm, *P. gingivalis* presented with increased adhesion, accompanied by a significant increase in cell viability, especially under normoxic conditions. These observations point to fungal biofilm as a source of potential receptors for *P. gingivalis* cell surface components and support the hypothesis that these biofilms form a protective environment for bacterial growth.

The preliminary gene expression analysis in our current study revealed that the expression of genes encoding the main fungal adhesins increased significantly under normoxia, whereas the aspartic protease-encoding genes, among which *SAP5* and *SAP6* have been identified as the major biofilm-related genes^41^, seemed to be mainly involved in fungal cell protection or defense against the bacterial neighborhood under anoxic conditions.

The following surface components of *P. gingivalis* were previously identified to be involved in cell-to-cell interactions: lipopolysaccharides (LPS), fimbriae, internalines and cysteine proteinases, i.e. gingipains^42^. Bacterial LPS can modulate *Candida* biofilm depending on the contacting bacterial species and contact duration^43^. Long fimbriae composed of FimA are mainly involved in co-aggregation with *Actinomyces viscosus*^44^, *Treponema denticola*^45^, *Streptococcus gordonii*^46^ and *Streptococcus oralis*^47^. However, no such interactions of FimA were detected in contact with *C. albicans* cells in a recent report^48^, where the authors presented also that *P. gingivalis* utilizes the InlJ internalin family protein to interact tightly with *C. albicans* hyphae within the biofilm structure. On the other hand, Kamaguchi *et al*. (2003) demonstrated that *P. gingivalis* vesicles, containing gingipains, strongly promote co-aggregation between *S. aureus* and filamentous form of *C. albicans* cells, suggesting a presence of a specific receptor for these proteins on the surface of both microorganisms and indicating a possible function of gingipains as bridging molecules in multispecies biofilms within the oral cavity^49^. These proteases, including arginine specific-gingipains, RgpA and RgpB, and a lysine specific gingipain Kgp are involved in hemagglutination^50^.

Within the structure of RgpA and Kgp, catalytic and hemagglutinin adhesive domains (Hgp44, Hgp15, Hgp17, Hgp27) have been identified, the latter of which are absent from RgpB^51^. The Hgp domains of gingipains are involved in the adherence to components of the intercellular matrix^52^ and in the binding of hemoglobin^50^ as well as in the binding and further activation of the human kinin-generating system^53^. Moreover, the importance of the Hgp44 domains of HRgpA and Kgp in the co-aggregation of *P. gingivalis* with *T. denticola* has also been documented^54^, as has the direct influence of Kgp on the composition and structure of a mixed biofilm with *Tanerella forsythia*^55^ and its indirect role in *A. actinomycetemcomitans* biofilm detachment^56^.

In our present experiments, we analyzed the differences in the interactions between wild type and gingipain-deprived *P. gingivalis* strains and concluded that the structure of gingipains, especially RgpA and Kgp, and the special contribution of their adhesive domains, could be particularly important for the cooperation with the *C. albicans* cell surface during biofilm formation. This hypothesis was additionally supported by our finding of a significant reduction of fungal cell viability under anoxia. Such close cell contact mediated by gingipains, which are proteins with both adhesive and proteolytic properties, could explain the observed changes in mixed biofilm characteristics.

During *C. albicans* hypha development within a biofilm, the structure becomes more prone to bacterial co-adhesion as the fungal cells expose several compounds on their surface that are susceptible to nonspecific, mostly hydrophobic or electrostatic interactions with bacteria. These surface molecules include, among others, surface located glucans and mannans, and also more deeply located chitin which will form an interaction if the bacteria approach the fungi closely enough^57^. Moreover, it has been reported that specific interactions with fungal mannoproteins can also occur depending on whether the nature of the relationship with bacteria is antagonistic or synergistic^38^. A good example of such a partner for synergistic interactions is Als3, a protein that is glycosylphosphatidylinositol-linked to the fungal cell surface and that belongs to the Als protein family uniquely expressed on hyphae. Its N-terminal region interacts with the SspB protein exposed on the surface of *S. gordonii* cells and has been thought to drive, at least in part, the formation of dual species biofilm^58^. Als3 has also been proposed to be a receptor for a *S. aureus* interaction that facilitates bacterial invasion into host tissues leading to systemic infections^59^.

In contact with *P. gingivalis* under anoxic and normoxic conditions, we here identified the involvement also of other proteins of the Als family, namely Als1–3. Surprisingly, the increased presence of these three proteins was detected mainly on the cell surface of fungi in contact with the *P. gingivalis* ∆K∆RAB mutant, with a lower surface expression observed when the *P. gingivalis* wild type strain was in contact. On the other hand, the Als3 binding capacity for wild type bacterial cells was found in our current analysis to be two-fold higher compared to the mutant strain, clearly indicating the involvement of gingipains in the interactions with Als3. This was further confirmed by the observation of complex formation between purified Als3 and RgpA, characterized by a low Kd (c.a. 10 nM), which was determined for the first time by our current analysis. Moreover, we could hypothesize that locally produced gingipains, being in close contact with fungal cells, can degrade the adhesins during prolonged mutual interactions. Such a conclusion was supported by our *in silico* analysis indicating many sites within Als3 structures that are prone to gingipain degradation. A degradation experiment we performed *in vitro* with purified proteins at optimal conditions also confirmed such an effect (data not presented). However, Als3 exposed on the fungal cell surface could be much less readily accessed and less sensitive to degradation due to glycosylation. Moreover, glycosylation of the Als proteins could also be responsible for its intriguing interactions with diverse types of bacterial proteins. Nevertheless, the role of the mannosyl portion of these proteins in the interaction with bacteria remains to be elucidated.

Another fungal cell wall protein overproduced during contact with *P. gingivalis* cells under both types of oxygen-availability conditions is mannoprotein Mp65, important for hyphal morphogenesis, membrane cell wall stability and organization, and, thus, biofilm formation. The RGD motif present in the Mp65 structure is directly involved in the adhesion properties of this protein^27^. In contact with *P. gingivalis*, we found that Mp65 was able to interact with RgpA at a similar affinity to Als3. Moreover, the analysis *in silico* and *in vitro* indicated a very definite stability of Mp65 against gingipain action (data not shown). However, it is also possible that Mp65 targets another or additional interaction partner(s) on the surface of the mutant bacterial strain.

Another fungal protein produced in increased amount upon contact with *P. gingivalis* cells, mainly under anoxic conditions but also, albeit marginally, under normoxia, was enolase. It is the most abundantly expressed cytosolic enzyme and is considered a multifunctional protein, with a predominant and classical role in the glycolytic pathway^60^ but also a factor that “moonlights” at the cell surface. As a surface protein, where it appears via a yet unknown translocation process, enolase was identified previously during *C. albicans* biofilm formation^26^ and was shown to be a partner for the interactions with human host proteins such as plasminogen and kininogen, mediate the interaction with extracellular matrix proteins like fibronectin, and promote a humoral immune response^25,61^. Recently, it was demonstrated that Eno1 at the fungal cell surface possesses transglutaminase activity, suggesting its involvement in the formation of covalent cross-links between cell wall proteins and chitin and/or glucan^62^, an effect that could stabilize a biofilm structure. Moreover, the role of bacterial enolase in interspecies interactions has been documented for *Lactobacillus jensenii* and *Neisseria gonorrhea*^63^. Our current study revealed the binding of Eno1 to both, mutant and wild *P. gingivalis* strains, suggesting the possibility that several bacterial partners exist for this protein. However, our more precise analysis of the binding between Eno1 and RgpA showed a 3-fold stronger affinity than that detected for the RgpA-Als3 complex. It could be suggested from this that the observed significant increase in enolase production, especially under anoxic conditions, is not only a metabolic response to an unfavorable environment but also has consequences for pathogen interactions. The increased enolase surface exposition did not correlate with previously detected expression of its encoding gene, probably owing to different timing of these events in our experiments or because the surface function of enolase can be regulated via more advanced processes that probably depend on many environmental stimuli. It is worth noting in this regard that surface-located fungal enolase is also vulnerable to citrullination by the peptidyl arginine deiminase produced by *P. gingivalis* with a possible consequence for protecting biofilm formation between *C. albicans* and *P. gingivalis*^64^.

Finally, an enzyme of interest that was identified from our present analysis to change its expression significantly on the fungal cells during contact with *P. gingivalis* was chitinase. This enzyme is associated with the yeast-to-hyphae transition and plays the role in the maintenance and architecture of *C. albicans* biofilm. Its altered presence in the mixed species biofilm suggests that the formation of these microbial communities is a diverse and dynamic process that attributes new properties to both pathogens and allows them to colonize new niches.

Our current analysis thus extends the understanding of *P. gingivalis* biofilm-driven chronic inflammatory disease that has been previously correlated with an increased risk of aspiration pneumonia, atherosclerosis, diabetes and rheumatoid arthritis^65^. Moreover, our findings in relation to the mutual interactions in biofilms that change over the contact time and respond to the host colonizing niche to protect the mutual coexistence of fungal and bacterial pathogens against antimicrobial therapy, creates new challenges in the future search for effective treatments of related diseases.

## Materials and Methods

### Bacterial growth conditions

*P. gingivalis* W83 (ATCC® BAA-308™) and gingipain mutants: ∆RgpB, lacking gingipain RgpB, and an isogenic gingipain-null mutant, ∆K∆RAB (lacking the presence of RgpA, RgpB and Kgp) were obtained as described previously^65^ and grown under anaerobic conditions (90% N_2_, 5% CO_2_, 5% H_2_) at 37 °C on blood agar plates or in liquid Schaedler broth supplemented with hemin (5 µg/ml;), L-cysteine (50 µg/ml), menadione (0.5 µg/ml), and additionally with tetracycline (1 µg/ml) in the case of ∆K∆RAB. Bacteria from the overnight cultures were centrifuged (4500 × g, 10 min) and the bacterial pellet was washed three times and resuspended in phosphate-buffered saline, pH 7.4 (PBS).

### Fungal growth conditions

*C. albicans* strain 3147 (ATCC^®^ 10231™) was purchased from American Type Culture Collection (Manassas, VA) and cultured aerobically at 30 °C on a YPD plate containing agar. The cells were then cultured for 4 h at 30 °C in YPD liquid medium on an orbital shaker (170 rpm) to achieve mid-logarithmic growth. The cells were then harvested by centrifugation (3000 × g, 5 min) and the cell pellet was washed twice with sterile PBS. *Saccharomyces cerevisiae* cells serving as a surrogate host for *C. albicans* Als3 (pBC542-*als3lg)* and *S. cerevisiae* Cwp1 (pBC542-*cwp1)*, as well as the BY4742 strain transformed with pBC542 were obtained and cultured as described previously^31^.

### Models of dual-species biofilm formation

Mixed species biofilm was prepared on the surface of flat-bottomed 96-well plastic plates (Corning Corporation, Corning, NY). In this present study, three different models of infection were applied to investigate *C. albicans* and *P. gingivalis* interactions. The term *sequential model* refers to surface infection, where one of the organisms plays a role as a first colonizer (*C. albicans* or *P. gingivalis*, respectively) and was grown overnight at optimal conditions after which the second coloniser was settled.

In *sequential model type I*, the *P. gingivalis* biofilm was first formed by settling the bacterial cells on the microplate wells (1 × 10^8^ cells/well) in Schaedler medium followed by an overnight incubation at 37 °C under anaerobic conditions maintained using a GENbox jar with a Genbox anaer generator (bioMérieux S.A., Marcy l’Etoile, France). *C. albicans* cells (5 × 10^5^) were then added and both organisms were cultivated for 3 and 24 hours at 37 °C under either normoxic or anoxic conditions. After this period, the supernatants were removed and each well was washed with PBS and further analysed as described below.

In *sequential model type II*, the suspension of *C. albicans* cells (5 × 10^5^ cells/well) in RPMI 1640 medium was added to each well of the 96-well plate and the fungal cells were grown overnight under normoxic conditions at 37 °C. Subsequently, *P. gingivalis* cells (1 × 10^7^ and 1 × 10^8^ cells/well) were added and both microorganisms were cultivated for 3 and 24 hours at 37 °C under normoxic or anoxic conditions. After gentle washing of the cells, the adhesion and microorganism viability within the biofilm were examined.

In the *simultaneous model*, *C*. *albicans* (5 × 10^5^ cells/well), and *P. gingivalis* (1 × 10^7^ and 1 × 10^8^ cells/well) cells were settled on the plate at the same time and competed for the surface under anoxic and normoxic conditions. After 3 and 24 h of coincubation, the unbound cells were removed by gentle washing of the plate with PBS and the formed biofilm was analysed as determined below.

In parallel with each of these mixed species biofilm models, monospecies biofilms were also developed under the same conditions to serve as an adequate reference.

### Staining of bacterial and fungal cells

Before the preparation of biofilm, *P. gingivalis* cells were labelled with CellTrace^TM^ CFSE staining probe (5(6)-carboxyfluorescein N-hydroxysuccinimidyl ester; Thermo Fisher Scientific, Waltham, MA) in the dark at 37 °C for 40 minutes. After washing out the excess unbound tracer, the labelled bacteria were used for biofilm formation. *C. albicans* cells were stained for 5 min with Calcofluor White solution (CFW, Sigma-Aldrich) at a final concentration of 1 µg/ml just prior to microscopic analysis or fluorescence measurements.

### Determination of microbial cell adhesion and viability

The adhesion of bacterial and fungal cells within the formed dual-species biofilms was examined as follow. After gentle washing with PBS, the fluorescence intensity of CFSE-labelled *P. gingivalis* cells at 390/440 nm (excitation/emission) and CFW stained *C. albicans* cells at 480/520 nm was determined using a Synergy™ H1 Microplate Reader (BioTek Instruments, Winooski, VT). To test the viability of biofilm-forming microbial cells, a colony-forming unit (CFU) assay was used. *C. albicans* cells were grown aerobically at 30 °C for 24 hours on YPD agar plates, whereas *P. gingivalis* cells were cultured anaerobically at 37 °C for 7 days on blood agar plates. After incubation, the numbers of colonies were counted in four replicates from each sample.

### Biofilm visualization with confocal scanning laser microscopy

The imaging of single- and dual-species biofilms was conducted as a Z-stack of images, using a Zeiss LSM 710 confocal laser scanning microscope set on Zeiss Axio Observer Z1 (Carl Zeiss, Oberkochen, Germany) with a 40× 1.4 NA oil immersion objective and with ZEN black version 8.10.484 software. For this purpose, 1 × 10^6^ *C. albicans* cells in 400 μl of RPMI 1640 were seeded into the chamber of Eppendorf Cell Imaging Coverglasses of 170 μm thickness (Eppendorf, Hamburg, Germany), coated with 0.05% poly-L-lysine and cultured under aerobic conditions at 37 °C for 16 hours to form the biofilm. After biofilm washing, 1 × 10^8^ CFSE-labeled *P. gingivalis* cells in 400 μl of RPMI 1640 were subsequently added to the chambers and further incubated at 37 °C for 24 hours under aerobic or anaerobic conditions, as described above. The biofilms were then stained with CFW and analyzed microscopically using an excitation wavelength of 405 nm to visualize CFW-stained fungal cells, and 488 nm to detect bacterial cells labeled with CFSE.

### Expression of genes encoding selected yeast virulence factors

After 3 hours of mutual contact between both microbes under anoxia and normoxia, total RNA was extracted with a TRI^®^Reagent (Sigma Aldrich) in accordance with the protocol provided by the manufacturer. For cDNA synthesis, each 20-µl reaction mixture consisted of 2 µg of RNA, 0.5 µg of oligonucleotide (dT)_18_ primer and 200 U of MLV reverse transcriptase (Moloney murine leukemia virus reverse transcriptase; Promega, Madison, WI). Quantitative PCR was performed with the SYBR green-based detection assay and the StepOnePlus real-time PCR system (Applied Biosystems, Waltham, MA). Per 10 µl of reaction mixture, 2 µl of cDNA, 0.2 µl of forward and reverse primers (10 µM) and 5 µl of SYBR KAPA master mix (Kapa Biosystems, Wilmington, MA) were used. PCR amplification was carried out using the primer sets specified in Table 2, with *ATC1* as a reference, and under the following conditions: 1 cycle of 95 °C for 10 min and 40 cycles of 95 °C for 20 s, 48–57 °C (depending on the analyzed gene) for 20 s, and 72 °C for 30 s. Samples were amplified in triplicate. Data analysis was performed using real-time PCR system Sequence Detection software (version 1.4; Applied Biosystems).

**Table 2:** Primer sets.

Gene	Primer sets	
*ALS3*	5′-TGCTGGTGGTTATTGGCAAC-3′	(FR)
	5′-GTCGCGGTTAGGATCGAATG-3′	(RV)
*ALS7*	5′-TGTTGGCCCTGTTTACAACG-3′	(FR)
	5′-ACCGGGACTGGCAATAGTAC-3′	(RV)
*SAP3*	5′-ATTCTCCAGGGTTTGTTGCTT-3′	(FR)
	5′-CCAGCTTGACATGAAACTTGAG-3′	(RV)
*SAP6*	5′- CCCGTTTTGAAATTAAATATGCTGATGG-3′	(FR)
	5′- GTCGTAAGGAGTTCTGGTAGCTTCG-3′	(RV)
*SAP9*	5′- ATGGTTCATTGGACATGACT-3′	(FR)
	5′- TTAAATCGTGGGACATAACC-3′	(RV)
*HWP1*	5′- TCAACTGCTCAACTTATTGCT-3′	(FR)
	5′- GCTTCCTCTGTTTCACCTTG-3′	(RV)
*ENO1*	5′- AAACCCAGAATCCGACCCATC-3′	(FR)
	5′- GACCCAAGCATCCCAGTCATC-3′	(RV)
*ATC1*	5′-GATTTTGTCTGAACGTGGTAACAG-3′	(FR)
	5′- GGAGTTGAAAGTGGTTTGGTCAATAC-3′	(RV)

### Identification of *C. albicans* surface-exposed proteins, after growth in single- or dual-species biofilms with *P. gingivalis*

Aliquots of 5 × 10^8^ *C. albicans* cells were grown with 5 × 10^9^ *P. gingivalis* W83 or ∆K/∆RAB cells in 10 ml of RPMI 1640 medium for 24 hours at 37 °C with gentle agitation, under normoxia or anoxia. Fungal surface-exposed proteins were analyzed as described earlier^64,66^, using cell surface shaving with trypsin and a shotgun proteomic approach, with a liquid chromatography-coupled tandem mass spectrometry (LC-MS/MS)-based protein identification. Protein ranking with an indicated fold change referring to the averaged normalized spectral abundance factors (NSAFs) was created after comparison of *C. albicans* surface-exposed proteins from cells grown in mixed-species biofilms with *P. gingivalis* with the sample containing fungal surface-exposed proteins of *C. albicans* grown in single-species biofilm.

### Microbial protein isolation and purification

*C. albicans* enolase was purified from yeast cells cultured in YPD medium at 30 °C for 16 hours, the cell wall mannoprotein 65 (Mp65) from post-culture supernatants of *C. albicans* cells grown in an amino acid liquid synthetic medium, pH 6.8^67^ at 37 °C for 72 hours and the agglutinin-like sequence protein 3 (Als3) from the whole mixture of candidal surface proteins extracted with β-1,6-glucanase (Takara Bio Inc., Otsu, Shiga, Japan) from the cell walls of *C. albicans* cultivated in hyphal forms in RPMI 1640 medium at 37 °C for 72 hours. A combination of ion-exchange chromatography (a Pharmacia Biotech Resource Q column, GE Healthcare, Uppsala, Sweden) and gel filtration (a Pharmacia Biotech Superdex 200 HR 10/30 column) was used for protein purification, as described previously^61^.

The purification of HRgpA was performed using the culture fluid from a HG66 strain of *P. gingivalis* according to the method described previously where gel filtration and arginine-Sepharose chromatography were applied^68^. Purified proteins were identified with LC-MS/MS analysis as described previously^69^.

### Analysis of the interactions between purified microbial proteins and *P. gingivalis* and *S. cerevisiae* cells

To evaluate the possible presence of bacterial receptors for fungal purified proteins **-** Als3, Mp65 and Eno1 - wild type *P. gingivalis* cells and mutant counterparts defective in gingipain production were suspended in RPMI medium at a density of 10^6^ cells/ml and incubated in the presence of FITC-labeled fungal proteins at a broad concentration range, for 1.5 h at 37 °C, under anoxic and normoxic conditions. After incubation, the *P. gingivalis* cells were washed three times with PBS and analyzed by flow cytometry (FACSCalibur, BD, NJJ). For each sample, 5 × 10^5^ cells were collected and analyzed using a 488 nm laser and FITC signal detector. The intensity of fluorescence was calculated using FlowJo 8.7 software.

To verify interactions between RgpA and the fungal adhesin Als3, the binding of 100 nM biotin-labeled RgpA for 1.5 hours at 37 °C to the *S. cerevisiae* cells (3 × 10^7^ yeast cell per tube) with heterologous expression of Als3 on the yeast cell surface was analyzed. This was compared to negative control *S. cerevisiae* transformed with pBC542 alone and yeast cells carrying the *CWP1* gene of *S. cerevisiae* in a pBC542 vector^31^. After incubation, the unbound material was washed out and the yeast cells were mixed with solution of streptavidin-conjugated horseradish peroxidase in PBS and incubated in the dark for 1 hour at room temperature. After washing, the cells were transferred to new tubes and the bound biotinylated bacterial protein was measured after treatment with 3,3′5,5′ tetramethylbenzidine as a substrate for HRP^70^.

### Determination of the binding parameters for complexes of bacterial (RgpA) and fungal (Als3, Mp65, Eno1) proteins using surface plasmon resonance (SPR) measurements

SPR measurements were performed at 25 °C using a Biacore 3000 (GE Healthcare, Milwaukee, WI) with a running buffer (RB) containing 10 mM HEPES, 150 mM NaCl, and 0.005% surfactant (v/v), pH 7.4. *C. albicans* proteins, Eno1, Als3 and Mp65 were immobilized onto a CM5 sensor chip (GE Healthcare) in accordance with the supplier’s instructions using a standard amine-coupling method. Immobilization was performed in 10 mM sodium acetate buffer to reach 300 resonance units (RU) for Eno1 and Mp65 (pH 4.0 and 4.,5, respectively), and 360 RU for Als3 (pH 4.0). To test the binding of *P. gingivalis* RgpA, solutions of this bacterial protein prepared in RB at concentrations of between 10–100 nM were injected over the chip surface containing the fungal protein of interest at 20 μl/min flow rate and with 2 minutes of contact time for the association and 2 minutes for the dissociation phase. The collected data were analyzed using BIA evaluation software version 4.1 (GE Healthcare). The dissociation and association rate constants (*k*_d_ and *k*_a_) and the equilibrium dissociation constants (*K*_D_) were calculated from the global fit of a simple (1:1) Langmuir model with a baseline drift.

## Data Availability

The datasets generated during and/or analyzed during the current study are available from the corresponding author on reasonable request.
